# Identification and Characterization of Pathogenic *Fusarium* Species Causing White Mold Disease in Cultivated Morels (*Morchella* spp.) in China

**DOI:** 10.3390/jof12030184

**Published:** 2026-03-04

**Authors:** Luzhen Wang, Qi Zhao, Muqing Bai, Yongwei Wang, Keling Liu, Rujia Liang, Frederick Leo Sossah, Odeshnee Naicker, Chunlan Zhang

**Affiliations:** 1 College of Landscape Architecture, Changchun University, Changchun 130022, China; wlz18043069795@126.com (L.W.); zq1716614933@126.com (Q.Z.); bai1254288900@126.com (M.B.); yongw0607@gmail.com (Y.W.); l13980509398@126.com (K.L.); a15866783681@126.com (R.L.); 2 Coconut Research Programme, Oil Palm Research Institute, Council for Scientific and Industrial Research (CSIR), Sekondi P.O. Box 245, Ghana; flsossah@gmail.com; 3 Department of Plant and Soil Sciences, University of Venda, Thohoyandou 0950, South Africa; odeshnee.moodley@gmail.com

**Keywords:** morels, *Fusarium* spp., white mold disease, pathogenicity, species diversity, China

## Abstract

White mold disease (WMD) is a major constraint to *Morchella* cultivation in China, leading to significant yield and quality losses. While *Fusarium* species are recognized plant pathogens, their diversity and role in WMD of morels have been poorly understood. This study aimed to identify and characterize *Fusarium* species associated with WMD in cultivated morels. Symptomatic ascocarps were collected from 22 cultivation bases across 16 provinces in China. A total of 120 *Fusarium* isolates were recovered and identified using morphological traits and multi-locus phylogenetic analysis. Twelve *Fusarium* species were identified, *F. acuminatum*, *F. avenaceum*, *F. clavum*, *F. compactum*, *F. falciforme*, *F. flocciferum*, *F. ipomoeae*, *F. mucidum*, *F. oxysporum*, *F. proliferatum*, *F. subglutinans*, and *F. verticillioides*, with most isolates recovered from northern China. Among them, *F. verticillioides* was the most common species (22.5%). Pathogenicity assays showed that all twelve of the identified *Fusarium* species were virulent to morel ascocarps. This is the first comprehensive report of these twelve *Fusarium* species causing WMD in morels, providing critical insights into pathogen diversity and virulence in *Morchella* production systems. These findings will support the development of targeted monitoring and management strategies to reduce the impact of WMD in morel cultivation.

## 1. Introduction

Morels (*Morchella* spp.), members of the order Pezizales, family Morchellaceae, are highly prized edible Ascomycetes renowned for their distinctive flavor, nutritional value, and medicinal properties [1]. These fungi thrive in cool, humid environments and are valued for their bioactive compounds, including amino acids, adenosine, vitamins, and polysaccharides [2,3]. Modern pharmacological studies have indicated antioxidant, anti-inflammatory, immunomodulatory, hepatoprotective, and hypoglycemic activities associated with morel consumption.

Given their high economic potential, the artificial cultivation of *Morchella* species has attracted growing global interest. A major breakthrough occurred in the 1980s when Ower (1982) successfully domesticated and produced morel fruiting bodies under controlled indoor conditions [4]. In China, field cultivation of morels began in 2012 [5] and has since expanded rapidly, reaching approximately 10,667 ha by 2021. The main cultivated species in China include *M*. *importuna*, *M*. *sextelata*, and *M*. *septimelata*, with *M. sextelata* accounting for over 85% of the national cultivation area. Morel production currently ranks 25th among the 29 commercially cultivated edible fungi in the country [6].

Despite its rapid expansion, large-scale morel cultivation faces significant challenges, particularly from fungal diseases that reduce both yield and quality. Each year, disease outbreaks affect approximately 25% of the total cultivation area. Several fungal pathogens have been implicated in morel diseases, including *Neonectria candida*, *Paecilomyces penicillatus*, and *Pseudodiploöspora longispora*, which are associated with white mold and pileus rot [7,8,9]. Other reported pathogens include *Fusarium incarnatum* (stipe rot), *Fusarium nematophilum* (stalk rot), and various species of *Clonostachys*, *Lecanicillium*, and *Trichoderma* [10,11]. Additionally, cobweb disease in morels has been attributed to *Cladobotryum* and *Hypomyces* species [12,13,14].

These diverse pathogens often produce dense, white mycelial growth on morel ascocarps, collectively described as white mold disease (WMD). Under favorable environmental conditions, particularly high humidity and temperature, WMD can spread rapidly, leading to severe production losses [8,15]. Currently, WMD is regarded as the most destructive disease affecting *Morchella* cultivation. Liu et al. (2023), using high-throughput ITS amplicon sequencing, identified *P. longispora* and *Clonostachys solani* as frequently occurring WMD pathogens [16]. However, metabarcoding has limitations, particularly in resolving closely related species. Thus, comprehensive pathogen identification using pure cultures and multi-gene phylogenetic analyses is needed.

**Figure 1:**
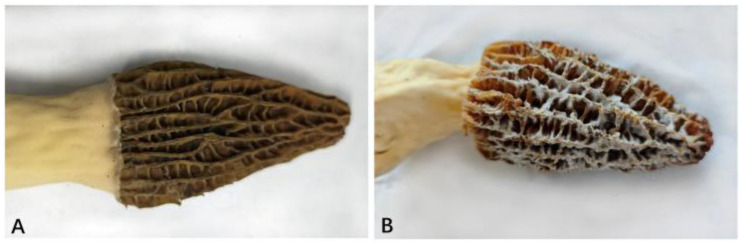
Fruiting bodies of cultivated *M*. *sextelata*. (**A**) Field-collected healthy ascocarp. (**B**) Field-collected fruiting body showing WMD symptoms.

To address these limitations and enhance understanding of the fungal pathogens associated with WMD in morels, in this study, we conducted a comprehensive investigation of *Fusarium* species affecting *M*. *sextelata* (Figure 1A). Specifically, the objectives of this study were as follows: (1) To isolate and identify *Fusarium* species associated with symptomatic morel ascocarps collected from diverse cultivation sites across China, using morphological and multi-locus phylogenetic analyses. (2) To evaluate the pathogenicity of representative isolates under controlled conditions. (3) To assess virulence variability among species to clarify their roles in WMD epidemiology. This study reveals the species diversity and pathogenic potential of *Fusarium* associated with diseases in morel cultivation. These results provide an important basis for the development of targeted prevention and control strategies for sustainable morel production.

## 2. Materials and Methods

### 2.1. Field Survey and Sampling

From 2023 to 2025, a field survey targeting WMD of morels was conducted across 22 cultivation sites in 16 major morel-producing provinces in China: Gansu, Heilongjiang, Hebei, Henan, Hubei, Inner Mongolia, Jiangsu, Jilin, Liaoning, Qinghai, Shaanxi, Shandong, Shanxi, Sichuan, Tianjin, and Yunnan. Sampling sites were selected based on the presence of symptomatic morel ascocarps exhibiting signs of WMD (Figure 1B). In total, 832 symptomatic ascocarps were collected. All samples were placed in sterile plastic bags, transported on ice, and stored at 4 °C prior to laboratory analysis.

### 2.2. Fungal Isolation and Culture

Fungal isolates were obtained using two complementary methods. For samples exhibiting sporulation, mature sporocarps (e.g., conidiophores, ascomata) were excised and suspended in sterile water to prepare spore suspensions. After serial dilution (10^−1^ to 10^−4^), 150 μL of each suspension was plated in triplicate on water agar (WA) supplemented with 100 μg/mL ampicillin and 100 μg/mL streptomycin to suppress bacterial contamination. After 24–36 h of incubation at 25 °C, germinated spores were individually transferred to potato dextrose agar (PDA) containing the same antibiotic concentrations (PDA-AS). Axenic cultures were established by subculturing single colonies onto fresh PDA.

For samples lacking visible sporulation, a tissue isolation method modified from Zhang et al. (2022) and Xiao et al. (2021) was used [17,18]. Sections (~3 × 3 mm) from the interface of healthy and diseased tissue were surface sterilized in 70% ethanol (1 min), followed by 1% NaClO (1 min), and rinsed three times in sterile distilled water. After drying on sterile filter paper, tissues were placed on half-strength PDA-AS and incubated at 25 °C. Emerging hyphal tips were transferred to PDA to establish pure cultures. All isolates and reference specimens were deposited at Changchun University Fungal Culture Collection (CCUCC), China.

### 2.3. Morphological Identification

Morphological characterization was conducted based on both macroscopic and microscopic features (Supplementary Table S1). Colony morphology, pigmentation, and growth rate were assessed after 7 days of incubation on PDA at 25 °C in the dark. Colony color was referenced using the Methuen Handbook of Colour [19]. Micromorphological features were examined using carnation leaf agar [20] and PDA, following the methods of Leslie and Summerell (2006) and Lombard et al. (2019) [21,22]. Observed traits included the presence and structure of sporodochia, conidiophores, phialides, aerial and sporodochial conidia, and chlamydospores. Samples were mounted in water and examined under a Carl Zeiss Axioscope 5 microscope (Carl Zeiss Microscopy GmbH, Oberkochen, Germany) equipped with Differential Interference Contrast (DIC) optics. Images were captured using a Carl Zeiss Axiocam 712 mono digital camera (Carl Zeiss Microscopy GmbH, Oberkochen, Germany), and at least 30 measurements were taken per structure using ZEISS ZEN 3.10 software.

### 2.4. DNA Extraction, PCR Amplification and Sequencing

Genomic DNA was extracted from 7-day-old cultures of each isolate grown on PDA at 25 °C using a plant DNA extraction kit (EE111-02; Beijing TransGen Biotech Co., Ltd., Beijing, China) according to the manufacturer’s instructions. Four genetic loci were targeted for amplification: the internal transcribed spacer (ITS) region using primers ITS4/ITS5 [23], calmodulin (*CaM*) using CL1/CL2A [24], translation elongation factor 1-alpha (*tef1*) using EF-1/EF-2 [25,26], and the second largest subunit of RNA polymerase II (*rpb2*) using fRPB2-5F2/7cR [27,28].

PCR reactions were conducted in a 50 µL volume containing 25 µL of 2× ExTaq PCR Mix (Sangon Biotech, Shanghai, China), 19 µL nuclease-free water, 0.5 µL of each primer (10 µM), and 5 µL of DNA template. Amplification conditions followed published protocols (Table 1). PCR products were verified by 1% agarose gel electrophoresis and sequenced by Sangon Biotechnology (Shanghai, China). Sequences were edited and assembled using PhyloSuite v1.2.3. All newly generated sequences were deposited in GenBank (Table 2).

### 2.5. Phylogenetic Analysis

Initial identification of all isolates was performed by comparing the ITS sequences against the NCBI GenBank database using BLASTn (https://blast.ncbi.nlm.nih.gov/Blast.cgi, accessed on 15 April 2025). For accurate species-level resolution, phylogenetic analyses were conducted on a subset of representative isolates using a multi-locus dataset comprising the internal transcribed spacer (ITS) region, the translation elongation factor 1-alpha (*tef1*), calmodulin (*CaM*), and the second largest subunit of RNA polymerase II (*rpb2*).

Reference sequences from type or ex-type strains of *Fusarium* species were retrieved from GenBank (Table 3) [29,30,31]. Multiple sequences (ITS, *tef1*, *CaM* and *rpb2*) were aligned using MAFFT within PhyloSuite v1.2.3 with auto strategy, and misalignments were corrected manually where necessary. Then, two independent phylogenetic algorithms, Maximum Likelihood (ML) and Bayesian Inference (BI), were employed for phylogenetic analyses according to the sequences (ITS, *tef1*, and *rpb2*) concatenated in PhyloSuite v1.2.3.

Specifically, phylogenetic analyses of TJS-16-2 and related strains were performed using a combined *CaM*, *rpb2*, and *tef1* dataset. The *CaM* gene sequence of TJS-16-2 was deposited in GenBank under accession number PQ595068. The CaM gene was selected because it is a core barcode for the identification of *Fusarium* species and provides high resolution for distinguishing closely related taxa, including strains such as TJS-16-2 that are prone to misidentification.

The Maximum-Likelihood (ML) analyses were carried out using MEGA X v10.2.6, with 1000 bootstrap replicates under the selected model tested using the MEGA X v10.2.6 software’s integrated Model Selection tool [32]. The Bayesian Inference (BI) analyses were performed using PhyloSuite v1.2.3, and modelFinder within PhyloSuite v1.2.3 was used to estimate the best-fitting model for sequences used in this study. For both the ML and BI analyses, the phylogenetic trees were viewed in MEGA X v10.2.6 and FigTree v1.4.4 (http://tree.bio.ed.ac.uk/software/figtree, accessed on 16 September 2025), respectively.

### 2.6. Pathogenicity Testing

To determine whether the 12 *Fusarium* species isolated from diseased *Morchella* ascocarps were all pathogenic, pathogenicity verification was carried out according to Koch’s postulates. One strain from each of the 12 *Fusarium* species was selected as a test isolate (representative isolates were selected based on geographic origin and colony morphology, Supplementary Table S2). Each isolate was grown on PDA for 14 days at 25 ± 1 °C in darkness to obtain active mycelial growth. Colonies were rinsed with sterile distilled water, suspensions were filtered through cheesecloth and adjusted to 1 × 10^6^ spores/mL using a hemocytometer and a 10 μL droplet of the spore suspension was applied onto the stipe surface. Sterile water served as the control. Each isolate was inoculated onto three independent ascocarps of *M. sextelata* (biological replicates), which were then transferred to an artificial climate chamber maintained at 18 °C and 90% relative humidity in darkness. Symptom development was monitored daily for 5 days post-inoculation. Lesion average diameter (mm) was measured using a digital caliper at 4 days post-inoculation.

To complete Koch’s postulates, symptomatic tissues were excised, surface-sterilized, and re-cultured on PDA [33]. The identity of re-isolated fungi was confirmed by morphological examination and sequencing of the internal transcribed spacer (ITS) region, following Zhang et al. (2021) [34]. All assays were conducted in triplicate, and the experiment was repeated twice to validate reproducibility.

### 2.7. Data Analysis

Pathogenicity data were subjected to one-way analysis of variance (ANOVA) using SPSS v27 (IBM Corp., Armonk, NY, USA). Prior to analysis, normality and homogeneity of variance tests were performed to verify the assumptions required for ANOVA. Post hoc comparisons of lesion sizes were conducted using Duncan’s multiple range test at *p* < 0.05. Because the sample size was equal across groups and the number of treatments was relatively large, Duncan’s test was selected for its efficiency and suitability for the present analysis. Graphical data (e.g., lesion size categorization) were visualized using Origin 2024 (OriginLab Corporation, Northampton, MA, USA).

## 3. Results

### 3.1. Symptom Description and Survey Findings

Symptoms observed in the field were consistent across sites and included dense, white, villous mycelial overgrowth, premature wilting, decay of ascocarps, and necrotic lesions (Figure 1B), in contrast to healthy ascocarps (Figure 1A).

**Figure 2:**
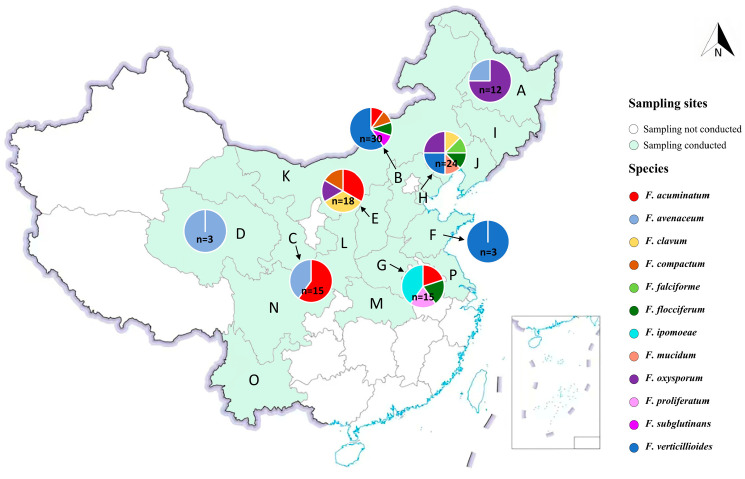
Sampling locations across China where *Morchella* ascocarps were collected for *Fusarium* isolation. Colors are used to distinguish *Fusarium* species. Letters A–P represent Heilongjiang, Hebei, Gansu, Qinghai, Shanxi, Shandong, and Henan provinces; Tianjin Municipality; Jilin, Liaoning, Shaanxi, Hubei, Sichuan, Yunnan, and Jiangsu provinces; and the Inner Mongolia Autonomous Region, respectively. Provinces marked in green on the map but without pie charts (i.e., locations I–P) indicate sites where no *Fusarium* was isolated from morel cultivation bases.

Based on field survey results and molecular identification, the presence and diversity of *Fusarium* species varied by region (Figure 2). Northern and north-central provinces, including Heilongjiang, Shaanxi, and Hebei, exhibited higher species richness and isolation frequencies compared with southern provinces. Isolation frequency was defined as the proportion of *Fusarium* species isolated from a single sampling location (province/municipality) relative to the total number of *Fusarium* species identified across all locations.

Other fungal genera isolated from the samples were distinguished from *Fusarium* based on macroscopic characteristics (e.g., mycelial morphology and colony color), microscopic features (e.g., macroconidia), and phylogenetic analysis using the ITS single-gene marker.

*Fusarium* species were isolated from morel cultivation bases in Heilongjiang, Hebei, Gansu, Qinghai, Shanxi, Shandong, and Henan provinces, as well as Tianjin Municipality. In contrast, no *Fusarium* isolates were obtained from Jilin, Liaoning, Shaanxi, Hubei, Sichuan, Yunnan, or Jiangsu provinces, or from the Inner Mongolia Autonomous Region, among the 16 provinces and municipalities surveyed (Figure 2). Pie charts at each sampling site illustrate the relative abundance of each *Fusarium* species identified. Notably, *F. acuminatum* and *F. verticillioides* were widely distributed and frequently isolated from multiple locations.

### 3.2. Molecular Identification

BLAST analysis of ITS sequences from 120 isolates obtained from symptomatic morel ascocarps revealed that all belonged to the genus *Fusarium*. To resolve species, the isolates were initially grouped based on colonial morphology and micromorphological characteristics. Representative isolates were then selected from each group by considering their geographic distribution, such that isolates from locations with higher isolation frequencies were proportionally represented. In total, 27 representative isolates were selected for multilocus phylogenetic analyses using ITS, *tef1*, *CaM*, and *rpb2* sequences. Reference species included in the phylogenetic analyses belonged to the same species complexes and exhibited high phylogenetic relatedness to the unidentified isolates based on *tef1* sequence comparisons, thereby improving the robustness of species delimitation.

**Figure 3:**
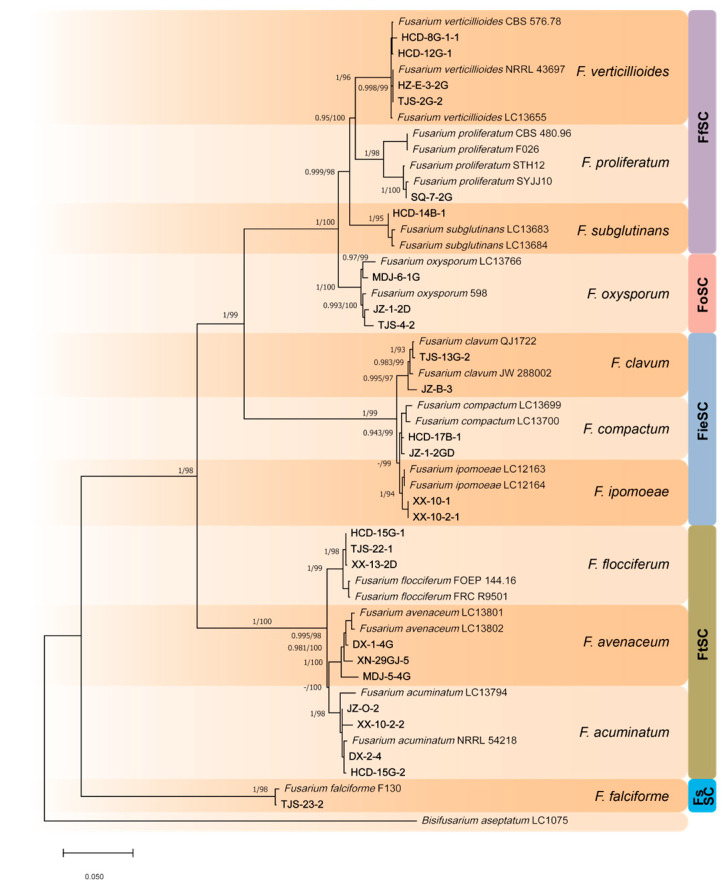
Phylogenetic tree based on combined ITS, *tef1* and *rpb2* gene regions for *Fusarium* pathogens causing WMD in *Morchella* (26 representative isolates) and related species. *Bisifusarium aseptatum* (LC1075) was used as the outgroup. Isolates from this study are shown in bold. RAxML bootstrap values (ML-BS > 70%) and Bayesian posterior probabilities (BI-PP > 0.9) are indicated at nodes (BI-PP/ML-BS).

**Figure 4:**
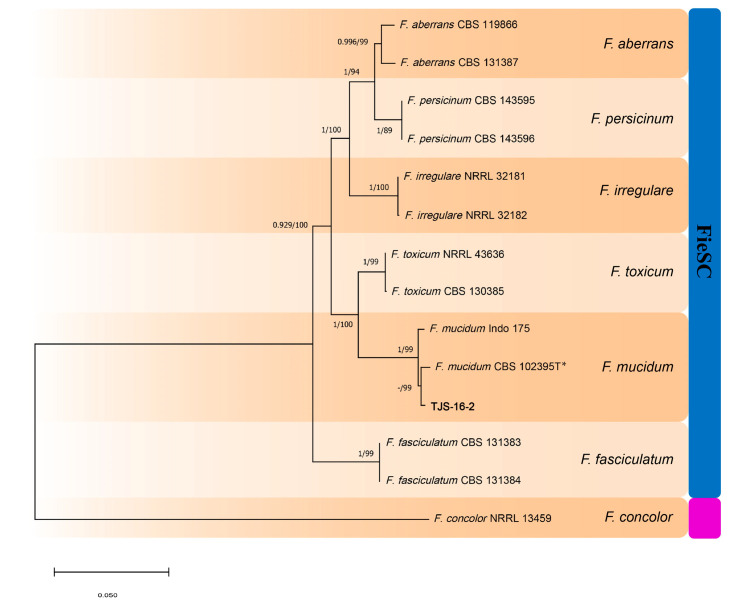
Phylogenetic tree based on combined *CaM*, *tef1* and *rpb2* gene regions for *Fusarium* pathogens causing WMD in *Morchella* (isolate TJS-16-2) and related species. *Fusarium concolor* (NRRL13459) was used as the outgroup. The isolate from this study is shown in bold. RAxML bootstrap values (ML-BS > 70%) and Bayesian posterior probabilities (BI-PP > 0.9) are indicated at nodes (BI-PP/ML-BS). * Ex-type strain.

The concatenated phylogenetic trees (ITS-*tef1*-*rpb2* and *CaM*-*tef1*-*rpb2*; Figure 3 and Figure 4) resolved 12 distinct *Fusarium* species: *F. acuminatum*, *F. avenaceum*, *F. clavum*, *F. compactum*, *F. falciforme*, *F. flocciferum*, *F. ipomoeae*, *F. mucidum*, *F. oxysporum*, *F. proliferatum*, *F. subglutinans*, and *F. verticillioides*. These species were grouped into five *Fusarium* species complexes, namely the *F. tricinctum* species complex (FtSC), *F. oxysporum* species complex (FoCS), *F. fujikuroi* species complex (FfSC), *F. solani* species complex (FsSC) and *F. incarnatum-equiseti* species complex (FieSC). High statistical support was observed at most nodes, with maximum likelihood (ML) bootstrap values ≥ 70% and Bayesian posterior probabilities (BPP) ≥ 0.90. *Fusarium concolor* NRRL 13459 and *Bisifusarium aseptatum* LC1075 were used as outgroups to root the trees.

### 3.3. Morphological Characteristics

**Figure 5:**
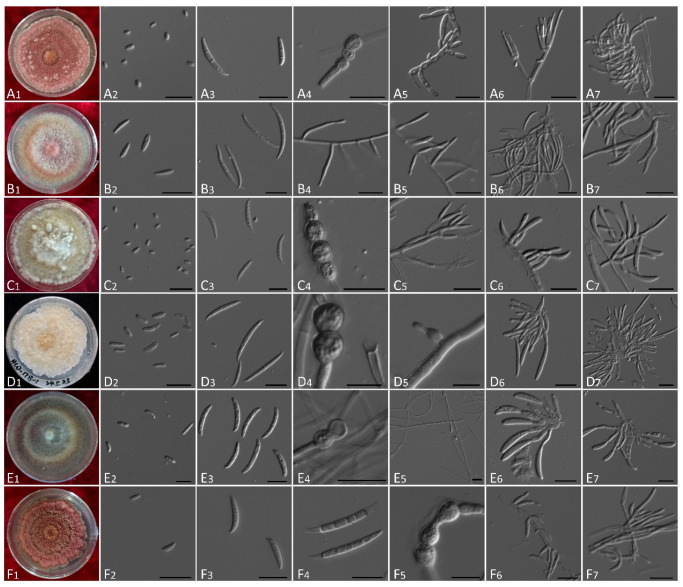
Morphological characteristics of six out of twelve *Fusarium* species identified. (**A**) *F. acuminatum*. (**B**) *F. avenaceum.* (**C**) *F. clavum.* (**D**) *F. compactum.* (**E**) *F. falciforme.* (**F**) *F. flocciferum.* (**A1**–**F1**), Colony on PDA. (**A2**–**F2**), Microconidia. (**A3**,**B3**,**C3**,**D3**,**E3**,**F3**,**F4)**, Macroconidia. (**A4**,**C4**,**D4**,**E4**,**F5**), Chlamydospores. (**A5**,**A6**,**B4**,**B5**,**C5**,**C6**,**D5**,**E5**,**F6**,**F7**), Aerial conidiophores. (**A7**,**B6**,**B7**,**C7**,**D6**,**D7**,**E6**,**E7**), Sporodochial conidiophores (not submerged). Scale bar = 20 μm (10 μm for (**D4**,**D5**,**F5**)).

**Figure 6:**
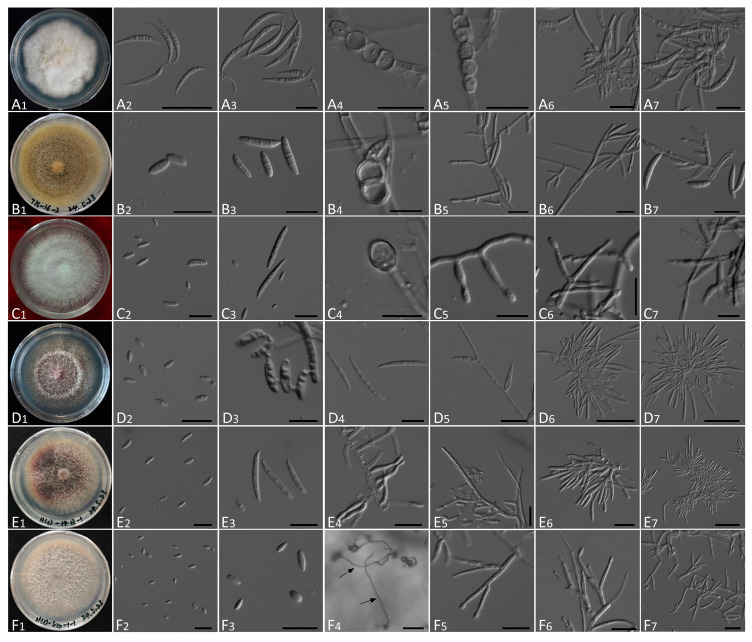
Morphological characteristics of six out of twelve *Fusarium* species identified. (**A**) *F. ipomoeae*. (**B**) *F. mucidum*. (**C**) *F. oxysporum*. (**D**) *F. proliferatum*. (**E**) *F. subglutinans*. (**F**) *F. verticillioides*. (**A1**–**F1**), Colony on PDA. (**B2**,**C2**,**D2**,**D3**,**E2**,**F2**,**F3**), Microconidia. (**A2**,**A3**,**B3**,**C3**,**D4**,**E3**), Macroconidia. (**A4**,**A5**,**B4**,**C4**), Chlamydospores. (**B5**,**B6**,**B7**,**C5**,**C6**,**D5**,**E4**,**E5**,**F5**,**F6**,**F7**), Aerial conidiophores. (**A6**,**A7**,**C7**,**D6**,**D7**,**E6**,**E7**), Sporodochial conidiophores (not submerged). Scale bar = 20 μm (10 μm for (**B4**,**C4**,**C5**,**C7**,**D3**); 50 μm for (**A2**,**D6**,**D7**,**E7**,**F4**)).

Morphological characteristics of the twelve *Fusarium* species isolated from symptomatic ascocarps of *M. sextelata* are summarized in Supplementary Table S1, with representative structures illustrated in Figure 5 and Figure 6. All isolates produced rapidly growing colonies on PDA, exhibiting diverse pigmentation and aerial mycelial textures ranging from sparse and villiform to dense and flocculent. All these characteristics were observed from strains cultured on PDA medium.

Colony features were generally consistent with descriptions of respective species in Leslie and Summerell (2006) [21]. For example, isolates of *F. acuminatum* and *F. flocciferum* formed compact, pinkish colonies with rose pigmentation, while *F. clavum* and *F. compactum* showed dense, flocculent growth and produced yellow to brown hues. In contrast, *F. oxysporum* and *F. proliferatum* colonies were villiform with violet pigmentation. Microscopically, all isolates produced typical fusarioid macroconidia, with the exception of *F. verticillioides*. Such spores were falcate to fusiform, hyaline, and varied in septation (1–7 septa). In addition, most species (excluding *F. ipomoeae*) could also produce oval to reniform microconidia from mono- or polyphialides. Notably, *F. verticillioides* and *F. proliferatum* produced chains of microconidia but lacked chlamydospores, while chlamydospores were abundant and diagnostic in species such as *F. acuminatum*, *F. mucidum*, and *F. compactum*. Sporodochial structures were observed in most species but lacking in *F. flocciferum* and *F. mucidum*.

Diagnostic traits distinguishing among species are detailed in Supplementary Table S1. Morphological observations were consistent with molecular identification and supported delimitation of *Fusarium* species.

### 3.4. Pathogenicity Tests

**Figure 7:**
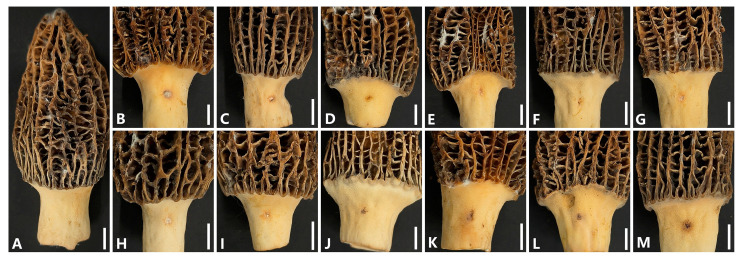
Pathogenicity of the twelve *Fusarium* species identified on *M. sextelata* ascocarps, 96 h post-inoculation. (**A**) Control. (**B**–**M**) Ascocarps inoculated with *F. acuminatum*, *F. avenaceum*, *F. clavum*, *F. compactum*, *F. falciforme*, *F. flocciferum*, *F. ipomoeae*, *F. mucidum*, *F. oxysporum*, *F. proliferatum*, *F. subglutinans*, and *F. verticillioides*, respectively. Scale bar = 10 mm.

**Figure 8:**
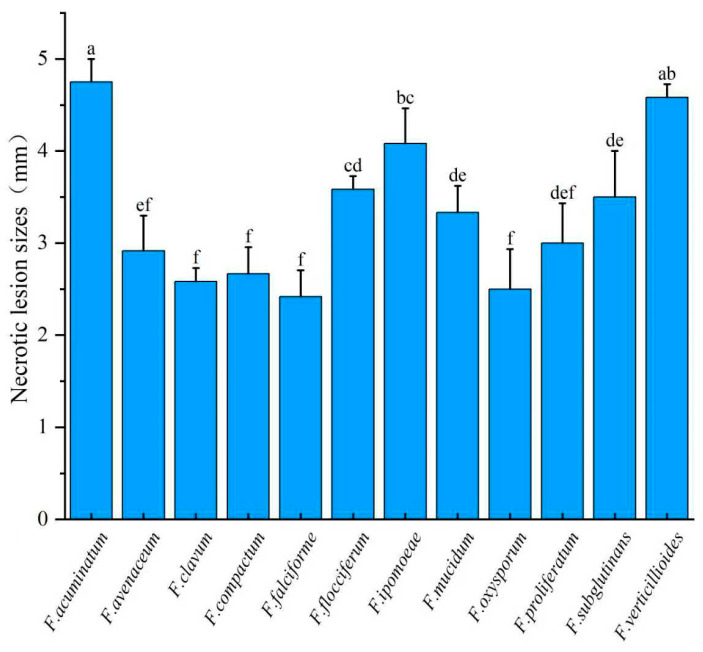
Necrotic lesion sizes on *M. sextelata* ascocarps 96 h after inoculation, showing the twelve *Fusarium* species identified. Data are means of three biological replicates. The different lowercase letters (a, b, c, d, e, f) above the bars indicate significant differences among treatments according to Duncan’s multiple range test at *p* < 0.05. Bars with the same letter are not significantly different.

Representative isolates of 12 *Fusarium* species were selected for pathogenicity assays (Supplementary Table S2). Isolates were considered virulent when obvious brown necrotic lesions developed near the inoculation sites on morel ascocarps shortly after inoculation, resulting in tissue damage. No obvious disease symptoms were observed within 1 to 2 days after inoculation. At 4 days post-inoculation, control ascocarps showed no symptoms (Figure 7A). In contrast, all twelve tested isolates induced distinct brown necrotic lesions with mean diameters ranging from 2.42 ± 0.29 to 4.75 ± 0.25 mm (*n* = 3) around the inoculation sites on morel ascocarps (Figure 7B–M). This demonstrated that all the identified *Fusarium* species were virulent to morel ascocarps (Figure 7 and Figure 8). All inoculated isolates were successfully re-isolated from symptomatic tissues, and their identity was confirmed via morphological and molecular methods. No fungi were recovered from the control group. These results fulfilled Koch’s postulates and confirmed the pathogenicity of all tested *Fusarium* species toward *M. sextelata*.

## 4. Discussion

Many members of the family Nectriaceae are known to infect fungal ascocarps, including species in the genera *Dialonectria* and *Neonectria*. The genus *Fusarium*, which also belongs to Nectriaceae, is large and ecologically diverse, encompassing species that function as saprotrophs, endophytes, or pathogens infecting a wide range of edible mushrooms and plant hosts [30,35,36]. *Fusarium incarnatum* was the first species reported to infect morels, and *F. nematophilum* was later confirmed as the causal agent of morel white mold disease (WMD). Other *Fusarium* species are known pathogens of edible mushrooms: *F. moniliforme* causes wilt disease in *Pleurotus ostreatus*, *F. solani* induces wilt disease in *Agaricus bisporus* and leads to yield losses, and *F. chlamydosporum* is responsible for white hair disease in *Auricularia auricula*. In addition, *Fusarium* species are among the most important pathogens of cereals and other crops, particularly wheat, maize, and soybean, posing major constraints to modern agricultural production [37,38,39,40]. Recent studies have further shown that some *Fusarium* species possess entomopathogenic potential [41], highlighting the broad pathogenic spectrum of this genus.

With the rapid expansion of *Morchella* cultivation, fungal diseases caused by *Fusarium* and other pathogens have become an increasing concern for producers. *Fusarium* species are widely distributed in nature; however, based on current research, only *F. incarnatum* and *F. nematophilum* have been identified as pathogens of morel ascocarps [29,42,43], and the involvement of other *Fusarium* species in WMD has remained poorly understood. To address this knowledge gap, we isolated 120 *Fusarium* strains from 832 diseased morel ascocarps and identified twelve *Fusarium* species associated with WMD in cultivated morels. This study represents the first report demonstrating that these twelve *Fusarium* species are pathogenic to cultivated morels.

Among the twelve pathogenic species identified, only *F. clavum*, *F. compactum*, and *F. ipomoeae* belong to the *F. incarnatum–equiseti* species complex (FieSC), together with the previously reported *F. incarnatum* and *F. nematophilum*. The remaining nine species were assigned to the *F. fujikuroi* species complex (FfSC), *F. oxysporum* species complex (FoSC), *F. solani* species complex (FsSC), and *F. tricinctum* species complex (FtSC). Unlike previous studies, which focused primarily on the FieSC, the present study demonstrates that *Fusarium* species from multiple species complexes beyond the FieSC can also cause WMD in morels, thereby expanding the known etiological diversity of this disease.

Morphological comparisons further supported the distinction between the *Fusarium* species identified in this study and previously reported pathogens of morels. In the study by Guo et al. [42], colonies of *F. incarnatum* were described as white to brown with slender, tapering macroconidia, characteristics that are partly shared with *F. clavum*, *F. compactum*, and *F. ipomoeae*. However, *F. incarnatum* has been reported to produce a distinct moldy odor, whereas no obvious odor was observed for *F. clavum*, *F. compactum*, or *F. ipomoeae* in the present study. In addition, microconidia are absent in *F. ipomoeae* [30], further differentiating it from *F. incarnatum*. In a previous study on stipe rot of *M. sextelata* caused by *F. nematophilum*, this species exhibited colony characteristics similar to *Pseudodiploospora longispora*, including white colonies with regular margins and distinct concentric rings [8]. In contrast, the *Fusarium* isolates obtained in this study produced pale rose, purple, or brown pigments, and none formed concentric rings, confirming that they are distinct from previously reported morel pathogens. Notably, *F. proliferatum* and *F. verticillioides* formed characteristic microconidial chains [44,45], and *F. mucidum* produced short, stout macroconidia. Furthermore, *F. avenaceum*, *F. subglutinans*, and *F. verticillioides* did not produce chlamydospores [46,47]. These morphological traits clearly distinguish the species identified in this study from *F. incarnatum* and *F. nematophilum*.

The frequent recovery of *F. verticillioides*, a well-known pathogen of maize and other cereals [48,49,50], suggests that certain *Fusarium* species possess strong ecological adaptability, enabling them to colonize diverse hosts, including morels. Although the precise mechanisms underlying WMD in morels remain unclear, and effective control strategies are still lacking, the results of this study highlight the importance of strict disease prevention, early detection, and integrated management in morel cultivation systems. Field investigations indicate that WMD occurrence is closely associated with high temperature and high humidity during the fruiting stage. In addition, large quantities of spawn and nutrient bags containing wheat and other plant-derived materials are commonly used in morel production. These nutrient bags are often prepared by farmers under relatively crude conditions, with inappropriate material selection and insufficient sterilization. Once grains become moldy or deteriorated, they may serve as reservoirs for pathogenic fungi, which can be introduced into cultivation soils and ultimately lead to WMD outbreaks.

To reduce the incidence of WMD, several measures are recommended, including the use of high-quality wheat grains and plant materials with thorough sterilization, maintenance of a clean production environment, regular monitoring of pathogen levels in soil and water sources, and optimization of cultivation systems to achieve effective control of temperature and humidity. When WMD symptoms are observed, priority should be given to collecting ascocarp samples showing typical symptoms at early disease stages to improve the success rate of pathogen isolation and identification.

Overall, this study demonstrates that multiple *Fusarium* species from diverse species complexes can cause WMD in morels, adding substantial complexity to disease management alongside other known pathogens such as *Pseudodiploospora*, *Clonostachys*, and *Paecilomyces*. The combined use of morphological characterization and multilocus phylogenetic analyses provides a robust framework for accurate pathogen identification and will facilitate the development of effective management strategies for WMD in *Morchella* cultivation. Future studies will focus on elucidating the biological characteristics of the isolated strains and exploring targeted prevention and control technologies based on these findings.

## 5. Conclusions

This study revealed a diverse range of *Fusarium* species associated with WMD in cultivated morels in China and clarified their pathogenic roles. Twelve species across five major *Fusarium* species complexes were identified, and all of these species exhibited virulence to morel ascocarps. These findings expand our understanding of the pathogens affecting *Morchella* and highlight the need for effective diagnostics and integrated management to reduce disease impacts in morel cultivation. Future research should explore how environmental and farming practices influence the spread and severity of these pathogens to support sustainable morel production.

## Figures and Tables

**Table 1:** Primers used in this study, including target loci, sequences, PCR programs, and references.

Gene/DNA Regions		Primers			PCR Amplification Procedures	References
Name	Abbreviation	Name	Direction	Sequence (5′–3′)		
Internal transcribed spacer region of the rDNA	ITS	ITS4	Forward	TCCTCCGCTTATTGATATGC	95 °C 5 min; 35 cycles of 95 °C 30 s, 52 °C 30 s, 72 °C 30 s; 72 °C 10 min; 10 °C soak	[23]
		ITS5	Reverse	GGAAGTAAAAGTCGTAACAAGG		
Calmodulin	*CaM*	CL1	Forward	GARTWCAAGGAGGCCTTCTC	95 °C 1 min; 35 cycles of 94 °C 30 s, 55 °C 30 s, 72 °C 30 s; 72 °C 10 min; 10 °C soak	[24]
		CL2A	Reverse	TTTTTGCATCATGAGTTGGAC		
RNA polymerase’s second-largest subunit	*rpb2*	5f2	Forward	GGGGWGAYCAGAAGAAGGC	94 °C 90 s; 35 cycles of 94 °C 45 s, 57 °C 45 s, 72 °C 30 s; 72 °C 5 min; 10 °C soak	[27,28]
		7cr	Reverse	CCCATRGCTTGYTTRCCCAT		
Translation elongation factor 1-alpha	*tef1*	EF-1	Forward	ATGGGTAAGGARGACAAGAC	94 °C 90 s; 30 cycles of 94 °C 45 s, 49 °C 45 s, 72 °C 30 s; 72 °C 10 min; 10 °C soak	[25,26]
		EF-2	Reverse	GGARGTACCAGTSATCATGTT		

**Table 2:** Details of 27 representative *Fusarium* strains isolated from cultivated *Morchella* in China.

Species	Voucher ID	Personal Numbers	GenBank Accession No.		
			ITS	*TEF*	*RPB2*
*F. acuminatum*	CCUCC 01137	JZ-O-2	PQ579876	PQ595067	PQ580797
*F. acuminatum*	CCUCC 00635	DX-2-4	PQ579867	PQ595063	PQ580792
*F. acuminatum*	CCUCC 01938	HCD-15G-2	PQ579864	PQ595058	PQ580790
*F. acuminatum*	CCUCC 00734	XX-10-2-2	PQ579871	PQ595064	PQ580794
*F. avenaceum*	CCUCC 00634	DX-1-4G	PQ579866	PQ595061	PQ580791
*F. avenaceum*	CCUCC 00832	MDJ-5-4G	PV549424	PV557318	PV557324
*F. avenaceum*	CCUCC 01235	XN-29GJ-5	PV549427	PV557319	PV557327
*F. clavum*	CCUCC 01134	JZ-B-3	PQ579875	PQ595066	PQ580796
*F. clavum*	CCUCC 02040	TJS-13G-2	PQ579855	PQ595048	PQ580788
*F. compactum*	CCUCC 01939	HCD-17B-1	PQ579865	PQ595051	PQ580784
*F. compactum*	CCUCC 01133	JZ-1-2GD	PV549423	PV557315	PV557323
*F. falciforme*	CCUCC 02045	TJS-23-2	PQ572449	PQ595049	PQ580775
*F. flocciferum*	CCUCC 02044	TJS-22-1	PQ579857	PQ595056	PQ580779
*F. flocciferum*	CCUCC 00735	XX-13-2D	PQ579872	PQ595059	PQ580795
*F. flocciferum*	CCUCC 01937	HCD-15G-1	PQ579863	PQ595060	PQ580783
*F. ipomoeae*	CCUCC 00732	XX-10-1	PQ579873	PQ595053	PQ580786
*F. ipomoeae*	CCUCC 00733	XX-10-2-1	PQ579874	PQ595054	PQ580787
*F. mucidum*	CCUCC 02043	TJS-16-2	PQ579856	PQ595065	PQ580778
*F. subglutinans*	CCUCC 01936	HCD-14B-1	PQ579862	PQ595050	PQ580782
*F. oxysporum*	CCUCC 01132	JZ-1-2D	PV549422	PV557314	PV557322
*F. oxysporum*	CCUCC 00833	MDJ-6-1G	PV549425	PV557316	PV557325
*F. oxysporum*	CCUCC 02047	TJS-4-2	PQ579854	PQ595047	PQ580777
*F. proliferatum*	CCUCC 00531	SQ-7-2G	PV549426	PV557317	PV557326
*F. verticillioides*	CCUCC 01943	HCD-8G-1-1	PQ579859	PQ595057	PQ580780
*F. verticillioides*	CCUCC 01934	HCD-12G-1	PQ579860	PQ595055	PQ580781
*F. verticillioides*	CCUCC 00332	HZ-E-3-2G	PV549421	PV557313	PV557321
*F. verticillioides*	CCUCC 02046	TJS-2G-2	PQ579853	PQ595046	PQ580776

Note: *F. acuminatum* CCUCC 01137 and *F. clavum* CCUCC 01134 had *M. importuna* as the host, and the remaining isolates were hosted by *M. sextelata*.

**Table 3:** Sequence information and details of related fungal strains retrieved from GenBank.

Species	Strain	GenBank Accession No.				References
		ITS	*TEF*	*RPB2*	*CaM*	
*F. aberrans*	CBS 119866	—	MN170444	MN170377	MN170310	[31]
*F. aberrans*	CBS 131387	—	MN170446	MN170379	MN170312	—
*F. acuminatum*	LC13794	MW016647	MW620108	MW474633	—	[30]
*F. acuminatum*	NRRL 54218	HM068326	HM068316	HM068336	—	—
*F. avenaceum*	LC13801	MW016655	MW620116	MW474641	—	[30]
*F. avenaceum*	LC13802	MW016656	MW620117	MW474642	—	[30]
*F. clavum*	JW 288002	MZ890484	MZ921826	MZ921694	—	—
*F. clavum*	QJ1722	PQ289631	PQ280811	PQ227791	—	—
*F. compactum*	LC13699	MW016526	MW594369	MW474512	—	[30]
*F. compactum*	LC13700	MW016527	MW594370	MW474513	—	[30]
*F. falciforme*	F130	OR123313	OQ511061	OR371857	—	—
*F. fasciculatum*	CBS 131383	—	MN170474	MN170407	MN170340	—
*F. fasciculatum*	CBS 131384	—	MN170475	MN170408	MN170341	—
*F. flocciferum*	FOEP 144.16	MN055985	MN061496	MN061498	—	—
*F. flocciferum*	FRC R9501	OL832290	OL772862	OL773166	—	—
*F. ipomoeae*	LC12163	MK280790	MK289597	MK289750	—	[30]
*F. ipomoeae*	LC12164	MK280822	MK289598	MK289751	—	[30]
*F. irregulare*	NRRL 32181	—	GQ505610	GQ505788	GQ505522	—
*F. irregulare*	NRRL 32182	—	GQ505611	GQ505789	GQ505523	—
*F. mucidum*	CBS 102395T *	—	MN170485	MN170418	MN170351	[29]
*F. mucidum*	Indo 175	—	LS479447	LS479862	LS479431	—
*F. oxysporum*	LC13766	MZ191879	MZ198890	MZ198892	—	[30]
*F. oxysporum*	598	PQ814364	PQ849378	PQ899757	—	—
*F. persicinum*	CBS 143595	—	LT970778	LT970750	LT970731	[31]
*F. persicinum*	CBS 143596	—	LT970779	LT970751	LT970732	[31]
*F. proliferatum*	CBS 480.96	—	MN534059	MN534272	—	—
*F. proliferatum*	F026	MZ379243	MZ399213	MZ399210	—	[30]
*F. proliferatum*	STH12	OR649272	OR663952	OR668954	—	—
*F. proliferatum*	SYJJ10	OR018534	OR047668	OR047671	—	—
*F. subglutinans*	LC13683	MW016510	MW580550	MW474496	—	[30]
*F. subglutinans*	LC13684	MW016511	MW580551	MW474497	—	—
*F. toxicum*	CBS 130385	—	MN170509	MN170442	MN170375	[31]
*F. toxicum*	NRRL 43636	—	GQ505663	GQ505841	GQ505574	[31]
*F. verticillioides*	CBS:576.78	KR071630	MW402142	KU604216	—	—
*F. verticillioides*	LC13655	MW01646	MW58050	MW47445	—	[30]
*F. verticillioides*	NRRL 43697	EF453174	EF453022	EF470061	—	—
*F. concolor*	NRRL 13459	—	GQ505674	GQ505852	GQ505585	[31]
*B. aseptatum*	LC1075	MW01638	MW580429	MW474375	—	[30]

Note: *F*. = *Fusarium*, *B*. = *Bisifusarium*; * ex-type sequence.

## Data Availability

The gene sequence data of the *Fusarium* strains used in this study were deposited in the NCBI GenBank database under the accession numbers: PV549421–PV549427, PQ579853–PQ579857, PQ579859–PQ579860, PQ579862–PQ579867, PQ579871–PQ579876 (ITS); PQ595046–PQ595051, PQ595053–PQ595061, PQ595063–PQ595067, PV557313–PV557319 (*RPB2*); and PQ580775–PQ580784, PQ580786–PQ580788, PQ580790–PQ580792, PQ580794–PQ580797, PV557321–PV557327 (*TEF1*). Other data are available upon request from the corresponding author.

## Supplementary Materials

The following supporting information can be downloaded at: https://www.mdpi.com/article/10.3390/jof12030184/s1, Table S1: Morphological characteristics of *Fusarium* species associated with white mold disease in *Morchella* in China; Table S2: Results of pathogenicity tests of the twelve representative *Fusarium* species identified on morel ascocarps.
