# Supplementary material for: Identification and Characterization of Pathogenic Fusarium Species Causing White Mold Disease in Cultivated Morels (Morchella spp.) in China
Source: J Fungi (Basel). 2026 Mar 4;12(3):184. doi: 10.3390/jof12030184 (PMC13028243; doi:10.3390/jof12030184)
Supplement: Supplementary file 1 [file jof-12-00184-s001.zip › Supplementary Table S1.pdf]

Supplementary Table S1. Morphological characteristics of *Fusarium* species associated with white mold disease in Morchella in China.

Characters	*F. acuminatum*	*F. avenaceum*	*F. clavum*	*F. compactum*	*F. falciforme*	*F. flocciferum*
**Macroconidia**						
Septate	2–4	3–6	1–6	1–5	1–4	1–6
Size (μm)	2-septate: 17.914–30.716 × 2.739–4.036 (av. 20.974 × 3.488), 3-septate: 20.618–33.441 × 2.801–4.050 (av. 24.813 × 3.497), and 4-septate: 33.834–36.463 × 4.053–4.596 (av. 35.149 × 4.325).	3-septate: 24.097–52.269 × 3.047–4.756 (av. 39.981 × 3.757), 4-septate: 41.258–57.120 × 2.911–4.316 (av. 48.251 × 3.663), 5-septate: 41.208–57.292 × 2.947–4.500 (av. 49.841 × 3.384), and 6-septate: 50.737 × 4.650 (n=1)	1-septate: 15.951–25.993 × 3.269–4.289 (av. 20.368 × 3.689, n=7), 2-septate: 19.137–36.291 × 3.531–4.666 (av. 25.380 × 3.969), 3-septate: 24.958–36.880 × 3.775–4.794 (av. 30.460 × 4.086), 4-septate: 32.238–40.146 × 3.432–4.771 (av. 35.886 × 4.247), 5-septate: 29.285–50.960 × 3.775–4.976 (av. 39.713 × 4.435), and 6-septate: 45.220 × 5.100 (n=1)	1-septate: 15.784–21.229 × 2.840–4.056 (av. 18.775 × 3.247, n=16), 2-septate: 18.655–24.401 × 3.113–4.848 (av. 21.522 × 3.560), 3-septate: 27.206–48.208 × 3.014–4.524 (av. 37.235 × 3.746), 4-septate: 30.249–36.592 × 4.025–4.580 (av. 31.740 × 4.330, n=5), and 5-septate: 32.556 × 4.696 (n=1)	1-septate: 20.379–36.703 × 4.053–6.255 (av. 24.155 × 4.967), 2-septate: 23.782–36.832 × 3.918–6.315 (av. 30.024 × 4.805), 3-septate: 29.421–42.916 × 4.449–5.556 (av. 35.778 × 5.037), and 4-septate: 37.497–45.670 × 4.615–7.984 (av. 41.648 × 5.947, n=8)	1-septate: 14.662–22.167 × 2.710–3.981 (av. 18.929 × 3.244), 2-septate: 18.120–26.343 × 2.454–3.954 (av. 20.633 × 3.187), 3-septate: 19.413–29.428 × 2.806–4.132 (av. 24.199 × 3.336), 4-septate: 31.352–51.675 × 4.295–5.554 (av. 38.243 × 4.873), 5-septate: 40.776–57.624 × 4.766–5.504 (av. 48.503 × 5.029, n=13), and 6-septate: 47.761–53.045 × 4.922–5.498 (av. 50.644 × 5.235, n=5)
Shape	Falcate, curved dorsiventrally, tapering towards both ends	Falcate, long, straight to slightly curved, with extended bent apical cells with indented basal cells	Falcate, curved dorsiventrally, tapering towards both ends, with a conical and curved apical cell and a blunt to foot-like basal cell	Fusiform to falcate, with strong dorsiventral curvature, apical cells elongated and tapering, and basal cellfoot-shaped	Falcate, curved, with well-developed foot cells and blunt apical cells	Fusiform or sickle-shaped, with two spiky ends
**Microconidia**						
Septate	0–1	0–2	0	0–1	0–1	0–1
Size (μm)	5.644–16.496 × 1.869–3.748 (av. 11.422 × 2.976)	7.981–23.926 × 2.133–4.370 (av. 11.992 × 2.766)	5.201–13.544 × 2.542–4.131 (av. 7.202 × 3.105)	7.546–17.699 × 1.893–5.119 (av. 10.669 × 3.207)	8.956–19.741 × 2.689–5.498 (av. 14.456 × 4.474)	3.925–11.197 × 1.587–3.023 (av. 5.573 × 2.122)
Shape	Oval, fusiform or reniform	Fusoid or reniform	Oval	Oval to ellipsoidal	Cylindrical, ellipsoid or reniform	Reniform, oval or fusiform
Chlamydospores (μm)	Usually formed in chains and clusters, 7.899–13.277 (av. 9.702) in diam	Absent	Abundant, terminal or intercalary, produced in pairs or chains, 7.894–11.952 (av. 9.718) in diam	Abundant, produced in chains or clusters, 7.603–14.803 (av. 10.560) in diam	Abundant, produced singly, in pairs, chains or clusters 6.776–11.497 (av. 9.043) in diam	Abundant, grew at the end or middle of the hypha, produced in chains or clusters, 6.012–13.441 (av. 9.451) in diam
**Conidiophores**						
Sporodochial conidiophores	Densely and irregularly branched, bearing apical whorls of 2–3 phialides	Densely and irregularly branched, bearing apical whorls of 2–4 phialides	Densely and irregularly branched, bearing apical whorls of 2–4 phialides	Densely and irregularly branched, bearing apical whorls of 2–5 phialides	Densely and irregularly branched, bearing apical whorls of 2–4 phialides	Absent
Sporodochial phialides (μm)	Subulate to subcylindrical, with lengths of 7.251–23.835 (av. 13.738), base widths of 1.716–3.396 (av. 2.705), and top widths of 0.882–1.788 (av. 1.421)	Subulate to subcylindrical, with lengths of 10.244–36.749 (av. 19.059), base widths of 1.305–2.617 (av. 2.631), and top widths of 1.195–1.936 (av. 1.553)	Subulate to subcylindrical, sometimes proliferating percurrently, with lengths of 8.424–17.519 (av. 12.108), base widths of 1.795–4.070 (av. 2.560), and top widths of 1.300–1.986 (av. 1.600)	Subulate to subcylindrical, with lengths of 13.434–24.992 (av. 17.407), base widths of 1.645–2.903 (av. 2.308), and top widths of 1.238–1.650 (av. 1.416)	Subulate to subcylindrical, with lengths of 12.181–24.330 (av. 16.504), base widths of 1.696–4.465 (av. 2.930), and top widths of 1.217–3.077 (av. 1.940)	Absent
Aerial conidiophores (μm)	Unbranched or penicillate branched, 10.764–126.804 × 1.855–4.607 (av. 42.970 × 2.703)	Unbranched or rarely branched, bearing terminal or lateral phialides, 14.153–43.217 × 1.774–2.915 (av. 28.932 × 2.299)	Unbranched or penicillate branched, 16.103–104.434 × 1.200–4.761 (av. 38.291× 2.540)	Unbranched or barely branched, boring terminal or lateral phialides, 7.896–21.651 × 1.760–3.343 (av. 12.056 × 2.464)	Unbranched or branched, 52.550–251.404 × 2.399–4.694 (av. 129.264 × 3.750)	Unbranched or irregularly branched, with 2–3 terminal phialides for some branches, 12.853–49.646 × 1.325–3.215 (av. 26.404 × 2.462)
Aerial phialides (μm)	Mono- and polyphialides, subulate to subcylindrical, with lengths of 9.028–26.142 (av. 15.269), base widths of 1.392–2.859 (av. 2.209), and top widths of 1.129–2.238 (av. 1.424)	Mono- and polyphialides, subulate to subcylindrical, with lengths of 10.758–38.914 (av. 21.019), base widths of 1.893–2.834 (av. 2.313), and top widths of 1.198–1.978 (av. 1.440)	Mono- and polyphialides, subulate to subcylindrical, with lengths of 10.245–29.414 (av. 16.487), base widths of 1.821–3.550 (av. 2.569), and top widths of 1.001–1.999 (av. 1.520)	Monophialidic, subulate to subcylindrical, with lengths of 5.087–10.395 (av. 7.649), base widths of 1.835–3.041 (av. 2.316), and top widths of 1.098–1.767 (av. 1.395)	Monophialides, long, cylindrical, often with collarette, and lengths of 44.806–192.381 (av. 92.424), base widths of 2.423–5.013 (av. 3.448), and top widths of 1.303–2.229 (av. 1.816)	Mono- and polyphialides, subulate to subcylindrical, with lengths of 9.346–33.123 (av. 14.635), base widths of 2.063–3.215 (av. 2.481), and top widths of 1.016–2.561 (av. 1.567)
**Colony characteristics**						
Surface	Floccose and compact, pinkish white to rose mycelia	Compact and villiform, white to pink, brown mycelia	Dense, felty to velvety, white, yellow to light brown mycelia	Dense, felty to velvety, white to light brown mycelia	Sparse and villiform, white to yellow mycelia	Dense and flocculent, pinkish white, pale rose and brown mycelia
Reverse	Rose and brown	White, pale gray rose and brown	White, yellow to light brown	White, ginger yellow to brown	White, yellow to light orange	Pink to rose
Growth rate (mm/d)	5.2	11.9	10.9	7	8.1	4.7

Supplementary Table S1 (cont.)

Characters	*F. ipomoeae*	*F. mucidum*	*F. oxysporum*	*F. proliferatum*	*F. subglutinans*	*F. verticillioides*
**Macroconidia**						
Septate	3–6	3–7	2–6	2–6	2–4	
Size (μm)	3-septate: 18.812–30.809 × 2.690–3.913 (av. 22.515 × 3.301), 4-septate: 27.009–36.471 × 3.031–4.545 (av. 31.959 × 3.796), 5-septate: 36.064–48.146 × 3.316–5.185 (av. 42.871 × 4.383), and 6-septate: 47.784 × 4.895 (n=1)	3-septate: 17 .946–31.344 × 3.767–5.451 (av. 24.455 × 4.503), 4-septate: 24.491–35.079 × 3.531–5.354 (av. 28.781 × 4.443), 5-septate: 28.229–39.507 × 3.573–5.364 (av. 33.313 × 4.358), 6-septate: 41.506 × 4.815 (n=1), and 7-septate: 41.286 × 5.260 (n=1)	2-septate: 26.987–36.338 × 3.885–4.111 (av. 31.390 × 4.001, n=4), 3-septate: 29.301–47.864 × 3.501–5.000 (av. 40.921 × 4.294), 4-septate: 42.738–56.957 × 3.619–5.532 (av. 48.353 × 4.513), 5-septate: 44.249–65.849 × 3.942–5.198 (av. 53.643 × 4.708, n=12), and 6-septate: 66.145–70.886 × 4.592–5.252 (av. 68.516 × 4.922, n=2)	1-septate: 12.876–20.409 × 2.368–3.388 (av. 17.303 × 2.812), 2-septate: 18.059–23.030 × 2.688–3.811 (av. 20.180 × 3.295), 3-septate: 20.112–37.659 × 2.776–4.268 (av. 31.280 × 3.577), 4-septate: 33.283–50.578 × 2.934–4.321 (av. 40.822 × 3.675), 5-septate: 37.728–54.584 × 3.254–4.380 (av. 47.313 × 3.676)	2-septate: 18.209–25.945 × 2.999–3.911 (av. 21.250 × 3.559, n=7), 3-septate: 22.419 × 2.957–4.890 (av. 34.000 × 3.875), and 4-septate: 38.595 × 3.204 (n=1)	Absent
Shape	Falcate, with strong dorsiventral curvature, smooth, and hyaline, with an apical cell hooked to tapering, basal cell foot-shaped	Falcate or fusiform, fat, straight or curved dorsiventrally, tapering towards both ends, with a blunt and curved apical cell and a blunt basal cell	Falcate, straight to slightly curved, relatively slender, with foot shaped to pointed basal cells and tapered and curved apical cells	Falcate, long, slender, straight to slightly curved	Falcate, straight or curved, hyaline, apical cell hooked to tapering, basal cell foot-shaped	
**Microconidia**						
Septate		0–1	0–2	0	0–1	0
Size (μm)	Absent	6.733–18.160 × 1.822–4.128 (av. 12.704 × 2.992)	5.603–18.556 × 2.321–5.249 (av. 9.885 × 3.395)	4.993–17.798 × 1.716–4.126 (av. 9.281 × 2.910)	6.357–15.781 × 2.244–4.129 (av. 10.773 × 3.082)	6.279–12.998 × 2.337–4.368 (av. 8.041 × 2.937)
Shape		oval or fusiform	oval, elliptical or reniform	ovoid or clavate,	oval	oval to club-shaped
Chlamydospores (μm)	Abundant, grew at the end or middle of the hypha, produced in chains or clusters, 7.351–13.226 (av. 10.198) in diam	Abundant, usually grew at the end of the hypha, produced in chains or clusters, 6.230–10.066 (av. 8.083) in diam	Abundant, produced singly or in pairs at the end of the hypha, and 5.605–9.807 (av. 7.820) in diam	Absent	Absent	Absent
**Conidiophores**						
Sporodochial conidiophores	Densely and irregularly branched, bearing apical whorls of 3–5 phialides	Absent	Irregularly branched	Densely and irregularly branched, bearing apical whorls of 2–4 phialides	Densely and irregularly branched, bearing apical whorls of 2–5 phialides	Absent
Sporodochial phialides (μm)	Subulate to subcylindrical, lengths of 5.388–12.572 (av. 8.872), base widths of 1.802–3.203 (av. 2.238), top widths of 1.020–1.739 (av. 1.332)	Absent	Subulate to subcylindrical, with lengths of 7.044–15.473 (av. 11.126), base widths of 1.695–3.362 (av. 2.374), and top widths of 0.852–2.025 (av. 1.404)	Ampoule-shaped, subulate or subcylindrical, with lengths of 8.900–16.029 (av. 11.659), base widths of 2.010–3.484 (av. 2.694), and top widths of 1.066–1.573 (av. 1.329)	Subulate to subcylindrical, with lengths of 11.236–30.057 (av. 19.609), base widths of 1.975–3.052 (av. 2.466), and top widths of 1.139–1.787 (av. 1.531)	Absent
Aerial conidiophores (μm)	Absent	Unbranched to sympodial or irregularly branched, bearing terminal or lateral mono- and polyphialides, 19.091–75.969 × 2.007–3.841 (av. 39.380 × 2.691)	Unbranched or a few verticillate branched, 11.835–47.363 × 1.936–3.184 (av. 23.318 × 2.448)	Rarely unbranched or irregularly branched, 15.225–120.924 × 1.885–3.495 (av. 50.708 × 2.566) in size,	Unbranched or verticillate branched, with apical whorls of 2–4 phialides for branched aerial conidiophores, 27.813–53.103 × 1.920–4.785 (av. 39.064 × 2.838)	Unbranched or verticillate branched, with apical whorls of 2–4 phialides for branched aerial conidiophores, 25.445–68.287 × 1.518–3.579 (av. 44.650 × 2.683)
Aerial phialides (μm)	Absent	Subulate to subcylindrical, sometimes proliferating percurrently, with lengths of 11.788–27.389 (av. 16.571), base widths of 1.466–3.720 (av. 2.497), top widths of 0.970–1.802 (av. 1.253)	Cylindrical, with lengths of 8.751–28.651 (av. 16.168), base widths of 1.839–3.145 (av. 2.404), and top widths of 1.195–1.967 (av. 1.568)	Subulate to subcylindrical, with lengths of 7.977–23.589 (av. 14.561), base widths of 1.348–3.013 (av. 2.161), and top widths of 0.793–1.309 (av. 1.078)	Subcylindrical, with lengths of 13.415–41.286 (av. 21.709), base widths of 1.647–3.489 (av. 2.539), and top widths of 1.063–1.914 (av. 1.429)	Subcylindrical, with lengths of 13.116–30.443 (av. 20.181), base widths of 1.926–3.044 (av. 2.385), and top widths of 0.984–1.736 (av. 1.445)
**Colony characteristics**						
Surface	Dense and flocculent, white and brown mycelia	Dense and villiform, white and brown mycelia	Sparse and villiform, white and pale violet mycelia	Sparse and villiform, white mycelia but may become purple-violet with age	Sparse and villiform, white pink, and purple mycelia	Sparse and villiform, white, pink, violet, light orange or
Reverse	White, light orange and brown	Yellow to brown	White and violet	White, violet to almost black	Purple and light bean paste	Light bean paste pigmentation with age
Growth rate (mm/d)	4.9	17	10.2	5.1	10.4	13.6

Note: All these characteristics were observed from strains cultured on PDA medium.
