# Supplementary material for: Identification and Characterization of Pathogenic Fusarium Species Causing White Mold Disease in Cultivated Morels (Morchella spp.) in China
Source: J Fungi (Basel). 2026 Mar 4;12(3):184. doi: 10.3390/jof12030184 (PMC13028243; doi:10.3390/jof12030184)
Supplement: Supplementary file 1 [file jof-12-00184-s001.zip › Supplementary Table S2.pdf]

Supplementary Table S2. Results of pathogenicity tests of the twelve representative *Fusarium* species identified on morel ascocarps.

**Species**	**Voucher ID**	**Lesion sizes（mm）**
F. acuminatum	CCUCC 00635	4.75 ± 0.25
F. avenaceum	CCUCC 00634	2.91 ± 0.38
F. clavum	CCUCC 02040	2.58 ± 0.14
F. compactum	CCUCC 01939	2.67 ± 0.29
F. falciforme	CCUCC 02045	2.42 ± 0.29
F. flocciferum	CCUCC 02044	3.58 ± 0.14
F. ipomoeae	CCUCC 00733	4.08 ± 0.38
F. mucidum	CCUCC 02043	3.33 ± 0.29
F. oxysporum	CCUCC 02047	2.50 ± 0.43
F. proliferatum	CCUCC 00531	3.00 ± 0.43
F. subglutinans	CCUCC 01936	3.50 ± 0.50
F. verticillioides	CCUCC 01934	4.58 ± 0.14
